# HPV Vaccination Training of Healthcare Providers and Perceived Self-Efficacy in HPV Vaccine-Hesitancy Counseling

**DOI:** 10.3390/vaccines10122025

**Published:** 2022-11-26

**Authors:** Ikponmwosa Osaghae, Charles Darkoh, Onyema Greg Chido-Amajuoyi, Wenyaw Chan, Paige Padgett Wermuth, Mala Pande, Sonia A. Cunningham, Sanjay Shete

**Affiliations:** 1 Department of Epidemiology, Human Genetics and Environmental Sciences, The University of Texas Health Science Center at Houston (UTHealth) School of Public Health, Houston, TX 77030, USA; 2 Department of Biostatistics, The University of Texas MD Anderson Cancer Center, Houston, TX 77030, USA; 3 Division of Cancer Prevention and Population Sciences, The University of Texas MD Anderson Cancer Center, Houston, TX 77030, USA; 4 Department of Epidemiology, The University of Texas MD Anderson Cancer Center, Houston, TX 77030, USA; 5 Department of Biostatistics and Data Science, UTHealth School of Public Health, Houston, TX 75207, USA; 6 Department of Management, Policy, and Community Health, UTHealth School of Public Health, Houston, TX 77030, USA; 7 Department of Gastroenterology, Hepatology and Nutrition, The University of Texas MD Anderson Cancer Center, Houston, TX 77030, USA

**Keywords:** Human papillomavirus, HPV vaccines, HPV vaccine-hesitancy, Provider’s self-efficacy, HPV provider training

## Abstract

HPV vaccine hesitancy is a key barrier to HPV vaccination. Using a population-based survey of HCPs practicing in Texas we determined the association between formal training of HCPs and perceived self-efficacy in counseling HPV vaccine-hesitant parents and adult patients. A total of 1283 HCPs completed the survey, with 879 providing vaccination services to pediatric patients and 1018 providing vaccination services to adult patients. Among HCPs included in this study, 405 of 577 (70%) and 315 of 505 (62%) perceived they were very/completely confident in counseling HPV vaccine-hesitant parents and adult patients, respectively. Compared to HCPs who received no training, those who received formal training in HPV vaccination promotion or counseling had 2.56 (AOR: 2.56; 95% CI:1.69–3.86) and 2.84 times higher odds (AOR: 2.84; 95% CI:1.87–4.33) of perceiving that they were very/completely confident in counseling HPV vaccine-hesitant parents and adult patients, respectively. Additionally, increasing years of practice and volume of patients seen were positively associated with being very/completely confident in counseling HPV vaccine-hesitant parents and adult patients. On the other hand, nurses were less likely than physicians to be very/completely confident in counseling HPV vaccine-hesitant parents. To increase HPV vaccination uptake, HCPs should receive tailored training to improve their self-efficacy in addressing HPV vaccine-hesitancy.

## 1. Introduction

Human papillomavirus (HPV) is the most common sexually transmitted infection in the United States (U.S.) [1]. Infection with low-risk HPV (types 6 and 11) are associated with benign skin lesions. In contrast, high-risk HPV types (e.g., types 16, 18, and others) are linked with six different cancers, including cervical, anal, penile, vaginal, and oropharyngeal [2]. The risk for various cancers attributable to HPV (types 6, 11, 16, 18, and others) ranges from 85% for cancers of the penis and vagina to 96% for cancer of the anus [2]. Moreover, infection with high-risk HPV types is considered a necessary but insufficient cause of cervical cancer [1,3]. The incidence rate of HPV-associated cervical cancer in Texas is 8.3 cases per 100,000 [4]. This rate is significantly higher than the U.S. average rate of 6.2 cases per 100,000 [4,5]. HPV-associated morbidity and mortality impose a substantial financial burden on individuals and governments.

There is overwhelming evidence to support the safety and efficacy of HPV vaccines for preventing HPV infection and associated diseases. To date, over 135 million doses of HPV vaccines have been administered in the U.S., with robust data from the Vaccine Adverse Event Reporting System (VAERS) confirming the safety of these vaccines [6]. Between 2015 and 2018, adverse event reporting decreased substantially.^7^ However, HPV vaccine hesitancy by parents and adult patients continues to undermine efforts aimed at increasing HPV vaccination uptake rates [7,8,9]. Even with increasing HPV vaccination recommendations by healthcare providers (HCPs), the coverage rates have remained suboptimal [10]. For example, the average HPV vaccination rate for teens aged 13–17 in 2020 was 59% nationally and 55% in Texas [11]. This rate is behind the national vaccination coverage rate for other childhood immunizations such as meningococcal, tetanus, diphtheria, pertussis (Tdap), measles, mumps, rubella (MMR), hepatitis B, and varicella vaccines [11].

HPV vaccine hesitancy is a significant barrier to HPV vaccination initiation and completion [12]. Studies have also found that providers’ perceived self-efficacy in their ability to counsel hesitant patients predicts HPV vaccination recommendation and uptake [13,14,15,16]. HCPs comfortable discussing HPV vaccination are over five-fold more likely to prescribe HPV vaccines [15]. However, most HCPs lack self-efficacy in providing confident recommendations to hesitant parents or patients [13,14,17]. Self-efficacy is a modifiable provider-level factor that could be enhanced through training and practice [18]. According to the self-efficacy theory, most individuals are capable of success if they acquire the right opportunities to develop the agency to pursue desired goals [19]. Improving the self-efficacy of HCPs through various training approaches is an essential step in achieving timely and strong vaccination recommendations and uptake [16,20,21,22]. However, there is limited data on the association between formal training of HCPs and their self-efficacy in counseling HPV vaccine-hesitant parents and adult patients. Therefore, this study aims to determine the association between the formal training of HCPs in HPV vaccination counseling and promotion and HCPs’ perceived self-efficacy in counseling hesitant parents and adult patients.

## 2. Methods

### Study Design, Data Source, and Population

This was a cross-sectional study based on the data from a state-wide HPV survey conducted by The University of Texas MD Anderson Cancer Center. The survey was administered online between January and April 2021. Email addresses of HCPs practicing in Texas were acquired from the LexisNexis Master Provider Referential Database [23]. HCPs currently practicing in Texas with an MD or equivalent degree (in the specialties of internal medicine, family medicine, obstetrics/gynecology, and pediatrics), physician assistants, or nurse practitioners with email addresses in the database were invited to complete a 10 min online survey. All participants provided informed consent. The study was approved by The University of Texas MD Anderson Cancer Center Ethical Review Board. Up to three email reminders were sent to non-respondents. Each participant was offered a $10 gift card as compensation for survey completion. This study is reported following the Strengthening the Reporting of Observational Studies in Epidemiology (STROBE) guidelines [24].

## 3. Measures

### 3.1. Dependent Variables

#### HCP’s Perceived Self-Efficacy or Confidence in HPV Vaccine-Hesitancy Counseling

The dependent variable was HCP’s perceived self-efficacy or confidence in knowledge and ability to counsel hesitant parents of pediatric (9–18 years) patients as well as adult (>18 years) patients. We had two dependent variables assessed separately using the following survey questions: How confident are you in your knowledge and ability to counsel: (1) Parents who are hesitant to vaccinate their child, (2) HPV vaccine-hesitant adult patients (>18 years)? Possible responses to both questions were “Not at all”, “Somewhat”, “Moderate”, “Very”, or “Completely.” Each dependent variable was operationalized as a binary variable, 0 = “Not at all/Somewhat/Moderate” and 1 = “Very/Completely”.

### 3.2. Independent Variable

#### 3.2.1. Training of HCPs

The primary independent variable was the formal training of HCPs. This was assessed based on the survey question, “Have you received formal training in HPV vaccination promotion or counseling (e.g., continuing medical education, workshops, and certified training seminars)?” This was a binary variable: yes versus no.

#### 3.2.2. HCP Socio-Demographic and Practice-Related Factors

The following HCP-related factors were also assessed: age (<35 years, 35–54 years, and ≥55 years), race/ethnicity (non-Hispanic White, non-Hispanic Black, non-Hispanic other, and Hispanic), HCP’s sex, and practice location (rural/urban). The zip code of primary work addresses reported by HCPs was used in determining their practice locations by linking the Federal Information Processing Standard (FIPS) Codes with the 2013 Rural-Urban Continuum Codes (RUCC) [25]. Other practice-related factors assessed include the number of years in practice (≤10 years, 11–20 years, and >20 years), practice type (solo practice, group practice, university or teaching hospital, Federally Qualified Health Center (FQHC)/public facility, and Other), the number of patients seen per week (0–50, 51–100, and >100), and provider type (physician, nurse, physician assistant, and other).

#### 3.2.3. Data Analysis

The distribution of formal training, socio-demographic and practice-related factors of HCPs was described across the strata of HCPs’ perceived self-efficacy or confidence in counseling HPV vaccine-hesitant patients using proportion and Pearson’s Chi-square test. This was presented separately for HCPs who reported perceived self-efficacy in counseling hesitant parents of pediatric patients and adult patients. We had two separate regression models for each dependent variable: HCPs who counsel parents and HCPs who counsel adult patients. We predetermined to include variables in our models based on the literature and relevance to our study question. Univariable and multivariable logistic regression analyses were used to estimate the odds of HCPs’ self-efficacy in counseling HPV vaccine-hesitant parents and adult patients while adjusting for all covariates included in the final model. We adjusted for HCPs’ age, sex, race/ethnicity, region of practice, years in practice, number of patients seen per week, role in practice, and facility type in each multivariable logistic regression analysis. Furthermore, we assessed the association between covariates and HCPs’ perceived self-efficacy in HPV vaccination hesitancy counseling. All analyses were performed using Stata/IC version 15.1. A two-sided *p*-value < 0.05 was considered statistically significant.

## 4. Results

A total of 1283 HCPs completed the online survey. The overall response rate was 7%, and there was no significant difference between respondents and non-respondents concerning provider type and sex of HCPs. Of the total HCPs who completed the survey, 614 (48%) practiced in facilities that served both adult and pediatric patients, 404 (31%) served only adult patients, and 265 (21%) served only pediatric patients.

### 4.1. HCPs’ Perceived Self-Efficacy in Counseling HPV Vaccine-Hesitant Parents of Pediatric Patients (9–18 Years)

Of the 879 HCPs who provided vaccination services to pediatric patients, 577 (66%) responded to the question on perceived self-efficacy in counseling HPV vaccine-hesitant parents of pediatric patients (9–18 years) (Table 1). Of these respondents, 405 (70%) HCPs perceived that they were very/completely confident in counseling HPV vaccine-hesitant parents compared to 172 (30%) who perceived that they were not at all/somewhat/moderately confident. HCPs who received formal training in HPV vaccination promotion or counseling were more likely to perceive that they were very/completely confident in counseling HPV vaccine-hesitant parents than those who received no training (79% vs. 59%). Additionally, HCPs ≥ 55 years of age (78%) were more likely to perceive that they were very/completely confident in counseling HPV vaccine-hesitant parents compared to those 35–54 years old (70%) and those aged <35 years of age (53%). Furthermore, physicians (74%) were more likely to perceive that they were very/completely confident in counseling vaccine-hesitant parents than physician assistants (67%) and nurses (63%). Additionally, HCPs with more than 20 years of practice (83%) and those who see >100 patients per week (81%) were more likely to perceive that they were very/completely confident in counseling vaccine-hesitant parents.

Results from multivariable logistic regression analysis (Table 2) showed that HCPs who received formal training in HPV vaccination promotion or counseling had 2.56 times higher odds (Adjusted Odds Ratio (AOR): 2.56; 95% CI: 1.69–3.86) of perceiving that they were very/completely confident in counseling HPV vaccine-hesitant parents compared to those who received no such training. Compared to physicians, nurses had 52% lower odds (AOR: 0.48; 95% CI: 0.29–0.80) of perceiving that they were very/completely confident in counseling HPV vaccine-hesitant parents. Additionally, compared to those with ≤10 years in practice, those with >20 years had 3.95 times higher odds (AOR: 3.95; 95% CI: 1.80–8.67) of perceiving that they were very/completely confident in counseling HPV vaccine-hesitant parents. Additionally, HCPs who saw 51–100 patients per week and those who saw >100 patients per week had 2.32 (AOR: 2.32; 95% CI: 1.48–3.63) and 3.97 (AOR: 3.97; 95% CI: 1.95–8.09) times higher odds, respectively, of perceiving that they were very/completely confident in counseling HPV vaccine-hesitant parents than those who saw ≤50 patients per week.

### 4.2. HCPs’ Perceived Self-Efficacy in Counseling HPV Vaccine-Hesitant Adult Patients (>18 Years)

Among the 1018 HCPs providing vaccination services to adult patients, 505 (50%) responded to the question on perceived self-efficacy in counseling HPV vaccine-hesitant adult patients (>18 years) (Table 1). Of these respondents, 315 (62%) HCPs perceived that they were very/completely confident in counseling HPV vaccine-hesitant adult patients, while 190 (38%) HCPs perceived that they were not at all/somewhat/moderately confident in counseling HPV vaccine-hesitant adult patients. HCPs who received formal training in HPV vaccination promotion or counseling were more likely to perceive that they were very/completely confident in counseling HPV vaccine-hesitant adult patients than those who received no training (74% vs. 49%). HCPs ≥ 55 years of age (70%) were more likely to perceive that they were very/completely confident in counseling HPV vaccine-hesitant adult patients compared to those 35–54 years of age (63%) and those aged < 35 years of age (42%). HCPs with more than 20 years of practice (77%) and those who see 51–100 patients per week (72%) were more likely to perceive that they were very/completely confident in counseling vaccine-hesitant adult patients.

Results from the multivariable logistic regression analysis (Table 3) showed that HCPs who received formal training in HPV vaccination promotion or counseling had 2.84 times higher odds (AOR: 2.84; 95% CI: 1.87–4.33) of perceiving that they were very/completely confident in counseling HPV vaccine-hesitant adult patients compared to those who received no training. Additionally, compared to HCPs with ≤10 years in practice, those with >20 years had almost five-fold higher odds (AOR: 4.72; 95% CI: 1.99–11.15) of perceiving that they were very/completely confident in counseling HPV vaccine-hesitant adult patients. Additionally, HCPs who saw 51–100 patients per week had 2.94 times higher odds (AOR: 2.94; 95% CI: 1.85–4.66) of perceiving that they were very/completely confident in counseling HPV vaccine-hesitant adult patients than those who saw ≤50 patients per week.

## 5. Discussion

In this population-based study, we evaluated the association between the formal training of HCPs in HPV vaccination promotion or counseling and their perceived self-efficacy in counseling vaccine-hesitant parents and adult patients. Our study revealed that about a third of the HCPs did not perceive themselves as having high self-efficacy in counseling HPV vaccine-hesitant parents or adult patients. The low self-efficacy seen in this study is consistent with previous studies, which also found a low confidence level among HCPs regarding HPV and HPV vaccines [13,14,26]. HCPs occupy a unique role in vaccination recommendation and uptake as patients tend to trust them when making health-related decisions such as receiving the HPV vaccination [27,28]. Providers’ lack of self-efficacy in discussing the HPV vaccine has been identified as a barrier to HPV vaccination promotion and uptake [13]. As such, our study’s low confidence level highlights the need to enhance the self-efficacy of HCPs to increase HPV vaccine uptake.

Importantly, our study found a positive association between the formal training of HCPs in HPV vaccination promotion or counseling and their perceived self-efficacy in counseling HPV vaccine-hesitant parents and adult patients. This finding aligns with studies that found an increase in provider self-efficacy following the implementation of HPV vaccination training interventions [29,30,31]. Of note, the effect of formal training on the self-efficacy of HCPs in addressing HPV vaccine hesitancy, as seen in our study, was consistent for both hesitant parents and adult patients. Whereas safety concerns top the reasons for HPV vaccine hesitancy among parents of adolescents, lack of knowledge and recommendation of HPV vaccine is the main reason for HPV vaccine hesitancy in adults [32,33]. Additionally, HPV vaccine initiation rates vary among adolescents and adults, indicative of potential disparity in the rate of hesitancy by patient age [34,35,36]. Therefore, our findings demonstrate the effectiveness of formal training in enhancing providers’ self-efficacy in addressing HPV vaccine hesitancy regardless of the patient’s age or reason for hesitation. Additionally, HCPs will benefit from various forms of communication training, including the use of evidence-based announcement strategies, customized scripts, decision aids, and fact sheets which have been found to facilitate discussions and recommendations of HPV vaccination [21,22,37,38]. Incorporating these approaches into HPV vaccination training of HCPs would increase their self-efficacy in addressing HPV vaccine hesitancy.

While our study found a similar level of perceived self-efficacy among nurses and physicians in counseling HPV vaccine-hesitant adult patients, nurses had a low perceived self-efficacy in counseling vaccine-hesitant parents compared to physicians. Undoubtedly, nurses remain key stakeholders in addressing vaccine-hesitancy. In the U.S., nurses often provide vaccination services and play an essential role in vaccine advocacy, counseling, and recommendation [13]. With increasing HPV vaccine hesitancy among parents, our findings indicate a valuable opportunity to implement tailored interventions to enhance nurses’ self-efficacy in counseling vaccine-hesitant parents [7]. Additionally, our study found a positive association between HCPs’ years in practice and the volume of patients seen with providers’ self-efficacy in counseling hesitant parents. This points to the essential role of experience in attaining self-efficacy. Resultantly, an HCPs’ self-efficacy in counseling HPV hesitant parents could be enhanced over the years through the persistence of counseling several hesitant parents [39]. Thus, HCPs should utilize every visit by hesitant parents as an opportunity to increase their self-efficacy in addressing HPV vaccine-hesitancy. Additionally, HPV vaccination training should target HCPs who are young in practice and see few patients as a way of minimizing missed opportunities to address the concerns of hesitant patients effectively.

Findings from this study are subject to some limitations. This is a cross-sectional study, and causality cannot be inferred. Additionally, this study may be prone to information bias from the subjective ascertainment of HCPs’ self-efficacy in counseling hesitant parents or adult patients. Although the response rate reported in our study was low, it is not uncommon to see low responses for online surveys involving HCPs, particularly in surveys of frontline HCPs, mostly in non-academic health settings [40]. However, this study is important as it highlights the impact of formal training on the self-efficacy of HCPs in addressing HPV vaccine hesitancy to increase HPV vaccination uptake nationally and in Texas. Additionally, this was a state-wide survey of frontline HCPs in Texas, increasing the generalizability of our findings.

## 6. Conclusions

In conclusion, about a third of HCPs had low perceived self-efficacy in counseling HPV vaccine-hesitant parents and adult patients. Nurses were less confident in counseling HPV vaccine-hesitant parents compared to physicians. Formal training of HCPs enhances their self-efficacy in addressing HPV vaccine hesitancy among parents and adult patients. Additionally, HCPs’ self-efficacy in counseling hesitant parents and adult patients increased with years of practice and counseling several hesitant patients. To increase HPV vaccination uptake, HCPs should receive tailored training in HPV vaccine hesitancy counseling, especially nurses and HCPs with few years in practice.

## Figures and Tables

**Table 1:** Distribution of training, demographics, and practice-related factors for HCPs by strata of HCPs’ perceived self-efficacy in counseling HPV vaccine-hesitant parents of pediatric patients and adult patients.

	HCPs’ Perceived Self-Efficacy in Counseling HPV Vaccine-Hesitant Parents of Pediatric Patients (9–18 Years) (*n* = 577)			HCPs’ Perceived Self-Efficacy in Counseling HPV Vaccine-Hesitant Adult Patients (>18 Years) (*n* = 505)		
HCP Characteristics	Not at All/ Somewhat/ Moderate (*n* = 172)	Very/ Completely (*n* = 405)	*p*-Value	Not at All/ Somewhat/ Moderate (*n* = 190)	Very/ Completely (*n* = 315)	*p*-Value
**Training, *n* (%)**			<0.001			<0.001
No	103 (41.4)	146 (58.6)		119 (51.5)	112 (48.5)	
Yes	68 (20.9)	257 (79.1)		70 (25.8)	201 (74.2)	
**Provider age, years, *n* (%)**			0.002			0.001
< 35	28 (46.7)	32 (53.3)		37 (57.8)	27 (42.2)	
35–54	108 (29.7)	256 (70.3)		112 (36.7)	193 (63.3)	
≥ 55	33 (22.3)	115 (77.7)		38 (29.7)	90 (70.3)	
**Sex, *n* (%)**			0.958			0.689
Female	128 (29.8)	302 (70.2)		140 (37.1)	237 (62.9)	
Male	42 (30.0)	98 (70.0)		47 (39.2)	73 (60.8)	
**Race/Ethnicity, *n* (%)**			0.375			0.013
Non-Hispanic White	79 (27.8)	205 (72.2)		83 (32.7)	171 (67.3)	
Non-Hispanic Black	13 (26.5)	36 (73.5)		24 (51.1)	23 (48.9)	
Hispanic	28 (30.8)	63 (69.2)		24 (32.4)	50 (67.6)	
Non-Hispanic Other	49 (35.8)	88 (64.2)		55 (46.2)	64 (53.8)	
**Practice location, *n* (%)**			0.49			0.053
Rural	7 (24.1)	22 (75.9)		6 (20.7)	23 (79.3)	
Urban	165 (30.2)	382 (69.8)		184 (38.7)	292 (61.3)	
**Provider type, *n* (%)**			0.048			0.455
Physician	89 (25.6)	259 (74.4)		84 (35.0)	156 (65.0)	
Nurse	52 (37.1)	88 (62.9)		63 (38.2)	102 (61.8)	
Physician Assistant	22 (33.3)	44 (66.7)		31 (40.8)	45 (59.2)	
Other	9 (39.1)	14 (60.9)		12 (50.0)	12 (50.0)	
**Type of practice, *n* (%)**			0.092			0.702
University/Teaching hospital	51 (39.2)	79 (60.8)		55 (40.4)	81 (59.6)	
Solo practice	16 (26.2)	45 (73.8)		19 (44.2)	24 (55.8)	
Group practice	69 (28.9)	170 (71.1)		66 (36.9)	113 (63.1)	
FQHC/Public facility	20 (25.0)	60 (75.0)		28 (35.0)	52 (65.0)	
Other	16 (23.9)	51 (76.1)		22 (32.8)	45 (67.2)	
**Years in practice, *n* (%)**			<0.001			<0.001
≤10 years	77 (39.5)	118 (60.5)		89 (47.1)	100 (52.9)	
11–20 years	63 (31.2)	139 (68.8)		65 (38.9)	102 (61.1)	
>20 years	31 (17.5)	146 (82.5)		33 (22.9)	111 (77.1)	
**No. of patients seen (per week), *n* (%)**			<0.001			<0.001
≤50	84 (43.1)	111 (56.9)		95 (49.5)	97 (50.5)	
51–100	68 (24.3)	212 (75.7)		70 (28.0)	180 (72.0)	
>100	17 (18.7)	74 (81.3)		20 (37.0)	34 (63.0)	

Missing observations for parents of pediatric patients: Training, 3; age, 5; sex, 7; race/ethnicity, 16; practice location, 1; years in practice, 3; number of patients seen, 11. Missing observations for adult patients: Training, 3; age, 8; sex, 8; race/ethnicity, 11; years in practice, 5; number of patients seen, 9.

**Table 2:** Bivariable and multivariable logistic regression analyses of the association between HCPs’ training, socio-demographic, and practice-related factors with HCPs’ perceived self-efficacy in counseling HPV vaccine-hesitant parents of pediatric patients (9–18 years).

HCP Characteristics	Crude OR (95% CI)	*p*-Value	Adjusted OR (95% CI)	*p*-Value
**Training**				
No	Ref	Ref	Ref	Ref
Yes	2.67 (1.85–3.85)	<0.001	2.56 (1.69–3.86)	<0.001
**Provider age, years**				
<35	Ref	Ref	Ref	Ref
35–54	2.07 (1.19–3.61)	0.01	1.57 (0.78–3.14)	0.205
≥55	3.05 (1.61–5.77)	0.001	0.91 (0.34–2.41)	0.843
**Sex**				
Female	Ref	Ref	Ref	Ref
Male	0.99 (0.65–1.50)	0.958	0.66 (0.40–1.09)	0.106
**Race/Ethnicity**				
Non-Hispanic White	Ref	Ref	Ref	Ref
Non-Hispanic Black	1.07 (0.54–2.12)	0.852	1.34 (0.60–3.00)	0.476
Hispanic	0.87 (0.52–1.45)	0.587	0.84 (0.46–1.51)	0.552
Non-Hispanic Other	0.69 (0.45–1.07)	0.097	0.73 (0.45–1.21)	0.225
**Practice location**				
Rural	Ref	Ref	Ref	Ref
Urban	0.74 (0.31–1.76)	0.491	0.74 (0.28–1.93)	0.533
**Provider type**				
Physician	Ref	Ref	Ref	Ref
Nurse	0.58 (0.38–0.88)	0.011	0.48 (0.29–0.80)	0.004
Physician Assistant	0.69 (0.39–1.21)	0.194	0.71 (0.36–1.38)	0.307
Other	0.53 (0.22–1.28)	0.159	1.05 (0.39–2.84)	0.926
**Type of practice**				
University/Teaching hospital	Ref	Ref	Ref	Ref
Solo practice	1.82 (0.93–3.55)	0.081	1.49 (0.68–3.27)	0.318
Group practice	1.59 (1.01–2.49)	0.043	1.19 (0.69–2.04)	0.534
FQHC/Public facility	1.94 (1.05–3.59)	0.036	1.95 (0.97–3.91)	0.062
Other	2.06 (1.06–3.99)	0.033	1.81 (0.85–3.88)	0.124
**Years in practice**				
≤10 years	Ref	Ref	Ref	Ref
11–20 years	1.44 (0.95–2.18)	0.084	1.20 (0.72–2.01)	0.488
>20 years	3.07 (1.90–4.98)	<0.001	3.95 (1.80–8.67)	0.001
**No. of patients seen (per week)**				
≤50	Ref	Ref	Ref	Ref
51–100	2.36 (1.59–3.50)	<0.001	2.32 (1.48–3.63)	<0.001
>100	3.29 (1.81–5.99)	<0.001	3.97 (1.95–8.09)	<0.001

OR: Odds Ratio; CI: Confidence Interval.

**Table 3:** Bivariable and multivariable logistic regression analyses of the association between HCPs’ training, socio-demographic, and practice-related factors with HCPs’ perceived self-efficacy in counseling HPV vaccine-hesitant adult patients (>18 years).

HCP Characteristics	Crude OR (95% CI)	*p*-Value	Adjusted OR (95% CI)	*p*-Value
**Training**				
No	Ref	Ref	Ref	Ref
Yes	3.05 (2.10–4.44)	<0.001	2.84 (1.87–4.33)	<0.001
**Provider age, years**				
<35	Ref	Ref	Ref	Ref
35–54	2.36 (1.37–4.08)	0.002	1.98 (1.00–3.93)	0.05
≥55	3.25 (1.74–6.06)	<0.001	0.72 (0.26–1.99)	0.522
**Sex**				
Female	Ref	Ref	Ref	Ref
Male	0.92 (0.60–1.40)	0.689	0.87 (0.52–1.46)	0.605
**Race/Ethnicity**				
Non-Hispanic White	Ref	Ref	Ref	Ref
Non-Hispanic Black	0.47 (0.25–0.87)	0.017	0.56 (0.27–1.18)	0.129
Hispanic	1.01 (0.58–1.76)	0.968	1.17 (0.62–2.20)	0.626
Non-Hispanic Other	0.56 (0.36–0.88)	0.012	0.61 (0.36–1.03)	0.066
**Practice location**				
Rural	Ref	Ref	Ref	Ref
Urban	0.41 (0.17–1.04)	0.059	0.41 (0.15–1.12)	0.081
**Provider type**				
Physician	Ref	Ref	Ref	Ref
Nurse	0.87 (0.58–1.32)	0.513	0.77 (0.47–1.28)	0.316
Physician Assistant	0.78 (0.46–1.33)	0.361	0.72 (0.38–1.36)	0.31
Other	0.54 (0.23–1.25)	0.15	0.77 (0.29–2.01)	0.589
**Type of practice**				
University/Teaching hospital	Ref	Ref	Ref	Ref
Solo practice	0.86 (0.43–1.71)	0.664	0.54 (0.24–1.23)	0.14
Group practice	1.16 (0.74–1.84)	0.519	0.76 (0.43–1.34)	0.34
FQHC/Public facility	1.26 (0.71–2.24)	0.428	0.94 (0.47–1.86)	0.85
Other	1.39 (0.75–2.57)	0.295	0.95 (0.47–1.92)	0.891
**Years in practice**				
≤10 years	Ref	Ref	Ref	Ref
11–20 years	1.40 (0.92–2.13)	0.121	1.01 (0.59–1.73)	0.966
>20 years	2.99 (1.85–4.85)	<0.001	4.72 (1.99–11.15)	<0.001
**No. of patients seen (per week)**				
≤50	Ref	Ref	Ref	Ref
51–100	2.52 (1.70–3.74)	<0.001	2.94 (1.85–4.66)	<0.001
>100	1.66 (0.90–3.10)	0.107	2.10 (0.98–4.50)	0.055

OR: Odds Ratio; CI: Confidence Interval.

## Data Availability

Data available from the corresponding author on reasonable request.
